# Supplementary material for: IFNγ-Treated Macrophages Induce EMT through the WNT Pathway: Relevance in Crohn’s Disease
Source: Biomedicines. 2022 May 8;10(5):1093. doi: 10.3390/biomedicines10051093 (PMC9139093; doi:10.3390/biomedicines10051093)
Supplement: Supplementary file 1 [file biomedicines-10-01093-s001.zip › biomedicines-1658882-supplementary.pdf]

biomedicines

Table S1. Primary antibodies used for Western blot analysis, Immunofluorescence and Flow Cytometry.

Antibody	Supplier	Dilution
βCATENIN	C2206, Sigma	1:1000
ECADHERIN	13-1700, Thermo Fisher	1:1000
CD45-FITC	560976, BD Biosciences	1:100
CD14-Brillant Violet 510	301842, Biolegend	1:100
CD64-PerCP-Vio700	130-101-422, Milteny Biotec	1:100
CD206-VioBlue	130-100-034, Milteny Biotec	1:100
CD16-PE	360704, Biolegend	1:100
CD86-PECY7	305422, Biolegend	1:100
IFNγ (AF1)	ab171081, Abcam	1:1000
TGFβ	ab215715, Abcam	1:1000
WNT2b	701856, Thermo Fisher	1:1000
WNT6	ab50030, Abcam	1:1000
SNAIL	ab180714, Abcam	1:1000
VIMENTIN	ab92547, Abcam	1:1000
Alexa Fluor 647 anti-Rabbit IgG (H + L)	A21443, Thermo Fisher	1:100
GAPDH	G9545, Sigma-Aldrich	1:5000

Table S2. Sequences of human primers used in real-time PCR.

Gene	Sense (5′–3′)	Antisense (3′–5′)	Fragment`s Size (bp)
IL1β	GCTCGCCAGTGAAATGATGG	TCGTGCACATAAGCCTCGTT	330
IL2	CAAACTCACCAGGATGCTCAC	TTCTACAATGGTTGCTGTCTCATC	247
IL4	AGCAGTTCCACAGGCACAAG	TCTGGTTGGCTTCCTTCACAG	108
IL6	AGTGAGGAAGCCAGAGC	ATTGTGGTTGGGTCAGGGG	143
IL8	AGAGACAGCAGAGCACACAAG	AATTTGGGGTGGAAAGGTTTGG	223
IL10	CCTGCCTAACATGCTTCGAG	TCTTGGTTCTCAGCTTGGGG	198
IL17	TAGTCCACGTTCCCATCAGC	CGGTCATTGCTCTCACTTGC	223
IFNγ	CGTTTTGGGTTCTCTTGGCTG	TCCGCTACATCTGAATGACCTG	99
TNFα	GCTGCACTTTGGAGTGATCG	GGGTTTGCTACAACATGGGC	138
CD16	GAAGGGGAAACCATCACGCT	GCAAACAGGAGGCACATCAC	293
CD86	GTCTGTCCACCCCATCAAC	GTATCACCAAAACCCCTCCC	114
CD206	CTTTGGACGGATGGACGAGG	CAAGGAAGGGTCGGATCGTG	201
CD44	CACACCCTCCCCTCATTCAC	TGGATGGCTGGTATGAGCTG	221
c-MYC	CCTTTGGGCGTTGGAAACC	GTCGCAGATGAAATAGGGCTG	115
COL1A1	CAGGCTGGTGTGATGGGATT	CAGGCTGGTGTGATGGGATT	317
COL1A2	AGAGAGCGGTAACAAGGGTG	GACCACGAGAACCAGGACTAC	155
E-CADHERIN	AACCCAAGCACGTATCAGGG	ACTGCTGGTCAGGATCGTTG	142
ENAH	GACACTTGTCTCCCGTCTCC	GTCTAGGCAATGTTGGCCCT	482
N-CADHERIN	TGTTTGACTATGAAGGCAGTGG	TCAGTCATCACCTCCACCAT	152
FSP1	GCACTTCCTCTCTCTTGGTCTG	AACTTCATTGTCCCTGTTGCTG	247
FGFR2	CCTGCGGAGACAGGTAACAG	GGTGTCTGCCGTTGAAGAGA	103
LGR5	TTCCACAGCAACAACATCAGG	CGAGGCACCATTCAAAGTCAG	159
αSMA	GTCCCAGACATCAGGGAGTAA	TCGGATACTTCAGCGTCAGGA	102
SNAIL1	AACTGCAAATACTGCAACAAGG	ATTCGGGAGAAGGTCCGAG	281
TGFβ1	GCGGACTACTATGCTAAAGAGG	TCAAAAGACAGCCACTCAGG	296
VIMENTIN	GCTCCTACGATTCACAGCCA	CGTGTGGACGTGGTCACATA	190
WNT2B	CGAGGCACCATTCAAAGTCAG	AGCCACCCCAGTCAAAGTCC	240
WNT6	CTGGGGGTTCGAGAATGTCAG	GGAACAGGCTTGAGTGACCG	165
β-ACTIN	GGACTTCGAGCAAGAGATGG	AGCACTGTGTTGGCGTACAG	57

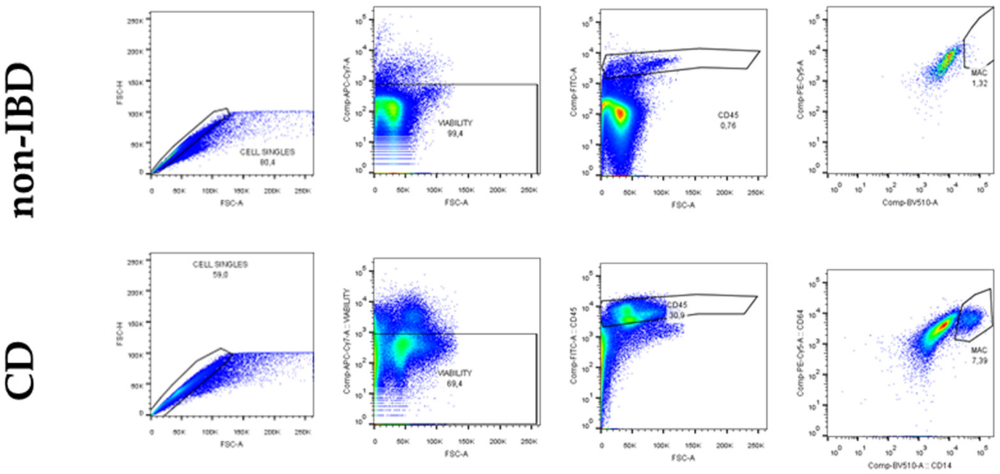

Figure S1. Identified *lamina propria* macrophages. Lamina propria macrophages were identified as single live CD45+ CD14+ CD64+ cells in cellular suspensions obtained from healthy mucosa of non-IBD group and from the affected mucosa of CD patients (*n* =7 per group).

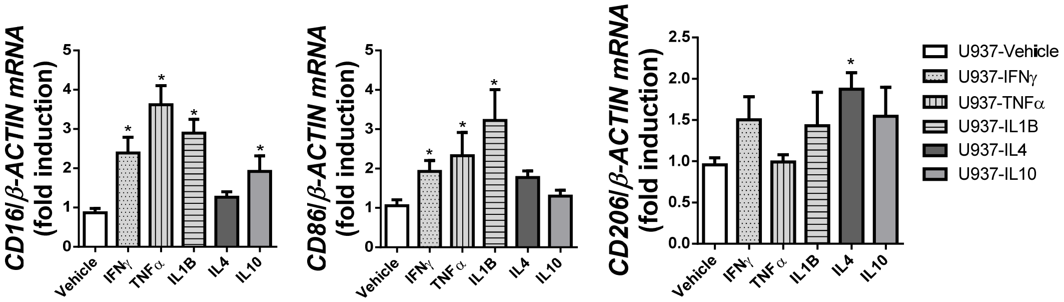

Figure S2. IFNγ-treated macrophages exhibited an increased CD86 and CD16 mRNA expression. U937 cells were treated with TNFα, IFNγ, IL1β, IL4 and IL10, or vehicle for 4 days. Graphs show the mRNA expression of CD16 (*n* = 6), CD86 (*n* = 6) and CD206 (*n* = 6). In all cases, bars in graphs represent mean ± SEM, and significant differences vs. U937-vehicle cells are shown by * $p < 0.05$.

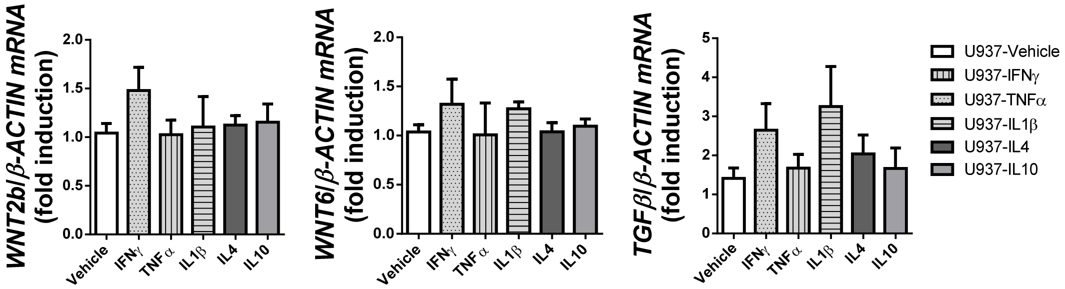

Figure S3. WNT and TGFβ mRNA expression in treated macrophages. U937 cells were treated with TNFα, IFNγ, IL1β, IL4 and IL10, or vehicle for 4 days. Graphs show the mRNA expression of WNT2b, WNT6 and TGFβ ($n \geq 3$ per group); In all cases, bars in graphs represent mean ± SEM.

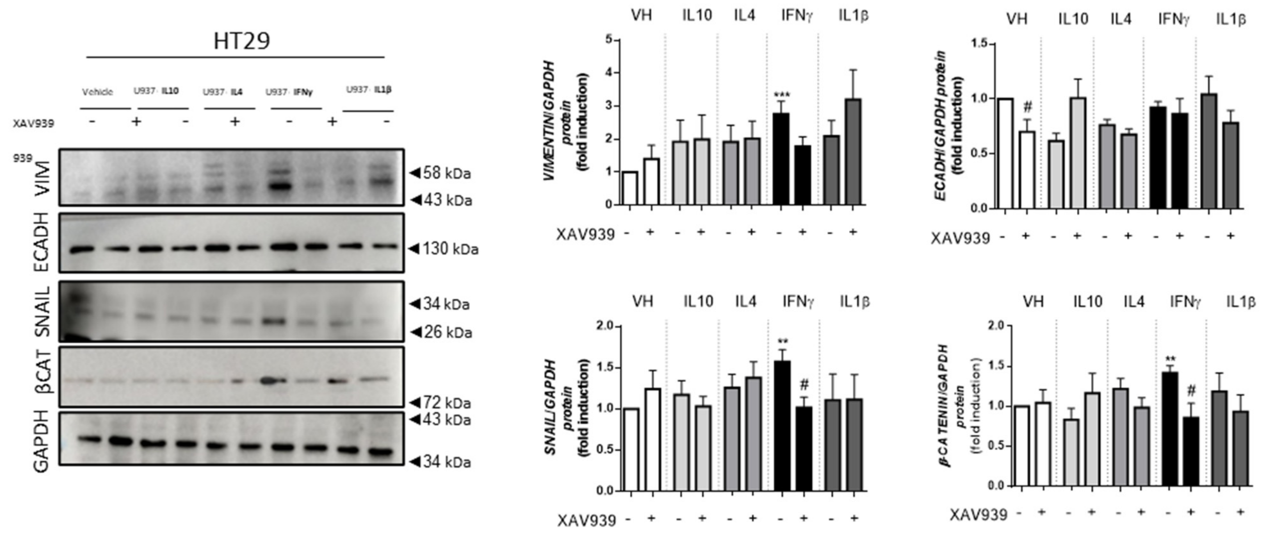

Figure S4. Treatment HT29 with XAV939 inhibits EMT process initiated by U937-IFNγ. HT29 cells were treated with XAV939 and then co-cultured for two 3 days with U937 cells, pretreated with IFNγ, IL10, IL4, or IL1β for 4 days. Graphs show the protein expression of EMT markers and βCATENIN in HT29 treated with XAV939 during the co-culture with U937cells. The images correspond to a representative Western blot ($n = 4$ per group). Bars in graphs represent mean ± SEM, and significant differences vs. HT29-vehicle cells are shown by ** $p < 0.01$ and *** $p < 0.001$; and vs HT29-vehicle-U937-IFNγ by # $p < 0.01$.
